# Lumbosacral plexopathy: A rare long term complication of concomitant chemo-radiation for cervical cancer

**DOI:** 10.1186/s40661-015-0019-9

**Published:** 2015-12-04

**Authors:** Imane Bourhafour, Meriem Benoulaid, Hanane El Kacemi, Sanae El Majjaoui, Tayeb Kebdani, Noureddine Benjaafar

**Affiliations:** Department of Radiotherapy, National Oncology Institute, Ibn Sina University Hospital, Mohamed 5 Souissi University, Rabat, Morocco

**Keywords:** Radiation Plexopathy, Lumbosacral plexus, cervical carcinoma

## Abstract

Radiation induced Lumbosacral plexophaty (RILP) is a rare but severe complication that has a considerable impact on quality of life. Its occurrence is rare but increasing with improved long-term cancer survival. This entity commonly results in different degrees of sensory and motor deficits. The pathophysiological mechanisms are not yet fully understood. Diagnosis of radiation myelopathy in women with gynecologic malignancies may increase with the use of concomitant chemo-radiation. This report describes the effect of this combination therapy in a 64-year-old woman with cervical carcinoma.

## Background

Cancer patients are surviving for many years and so the management of late treatment related complications that reduce their quality of life has become a public health priority [1].

One of the complications associated with pelvic radiation that is extremely rare but, at the same time, provides many diagnostic and even more therapeutic problems is radiation- induced lumbosacral plexopathy (RILP) [1, 2]. It is a chronic handicap, frightening because progressive and usually irreversible, usually appearing several years after radiotherapy [1]; diagnosis is by exclusion and the treatment remains a matter of development thanks to the better understanding of its pathophysiology. Its occurrence is rare but increasing with improved long-term cancer survival.

We present a case of cervical cancer patient successfully treated, who developed nine years after, serious radiotherapy-induced complications in the form of lumbosacral plexopathy.

## Case Report

A 64-year-old woman, G16 P12, was admitted in December 2004, with an 8-month history of pelvic pain associated with minimal post coital bleeding, his Medical history was unremarkable. Examination was significant for a 3 cm, exophytic cervical lesion; biopsies confirmed a differentiated squamous cell carcinoma. Patient preferred to have a radical hysterectomy. Surgical staging revealed a 6 cm of differentiated squamous cell cervical carcinoma with proximal left parametrial invasion, but no significant adenopathy, compatible with stage IIB.

Following tumour board discussion the patient was scheduled to receive external radiation to the pelvis followed by brachytherapy to the tumor mass. Along with radiation, the patient was planned to receive Cisplatin, at the dose of 40 mg per square meters per week (mg/m^2^/week).

In the period from 4 February 2005 to 16 March 2005, radiotherapy was delivered with once daily treatment at 2 Gy per fraction per day, five fractions per week. The overall schema is to the small pelvis for 23 fractions to 46 Gy.

Linear accelerators with beam energy of 25 MV were used. The small pelvis field included an anteroposterior/ posteroanterior fields to encompass 3 to 4 cm of the vaginal cuff, the parametria, the entirety of the external iliac, internal iliac, and obturator nodal basins. The field borders were as follows: superior at the L4-L5 interspace, laterally 1.5 cm beyond the bony pelvis, and inferior at the obturator foramen. Concurrent with radiation, five courses of Cisplatin (40 mg/m^2^/week) were administered; the treatment was tolerated well with decrease in pelvic pain, a urinary tract infection treated with oral antibiotics, and intermittent diarrhea.

Six weeks following the completion of radiation, an additional 15 Gy were administered with LDR brachytherapy using a vaginal cylinder applicator loading with Iridium 192 source, the dose is delivered at 0.25 to 1 Gy/h per daily fraction. The total dose treatment was 61 Gy and the total duration was approximately 13 weeks.

Follow-up examination included bimanual examination every 3 months for 2 years, with abdominal and pelvic CT when indicated, every 6 months for 3 more years and yearly thereafter.

Nine years after completion of treatment, the patient was readmitted due to progressive, bilateral leg pain and lower extremity weakness, greater on the left side, leaving her unable to walk, with sensory changes over the lateral legs and feet, her bladder and rectal functions remained intact.

Physical exam was significant for considerable remission of the central tumor with some radiation changes in the vaginal part. Neurologic exam found a bilateral lower extremity weakness greater on the left side, with decreased motor function in hip and knee flexion, weakening of pain and touch sensations in respect to L5, S1 roots.

**Fig. 1:**
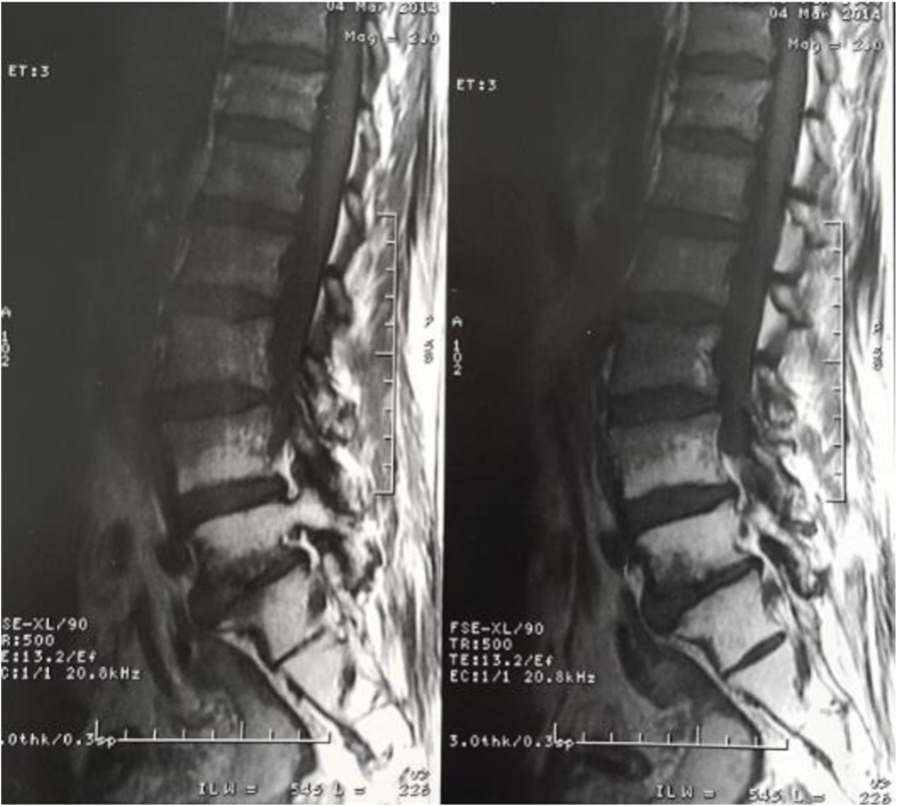
Spinal cord MRI T1-weighted sequence

**Fig. 2:**
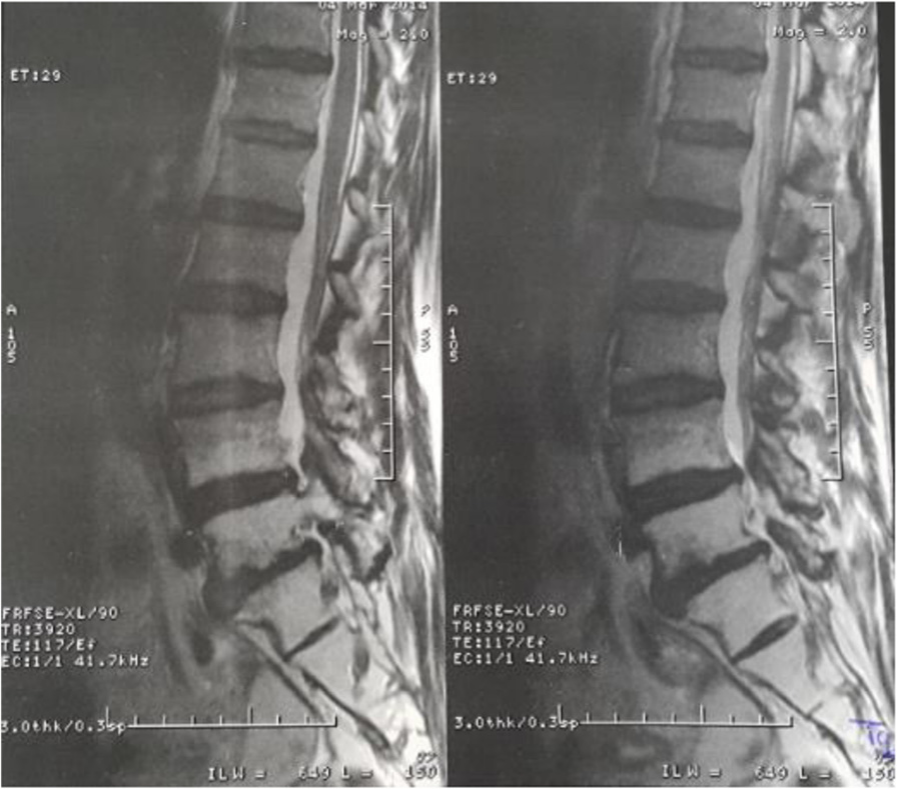
Spinal MRI T2-weighted sequence

**Fig. 3:**
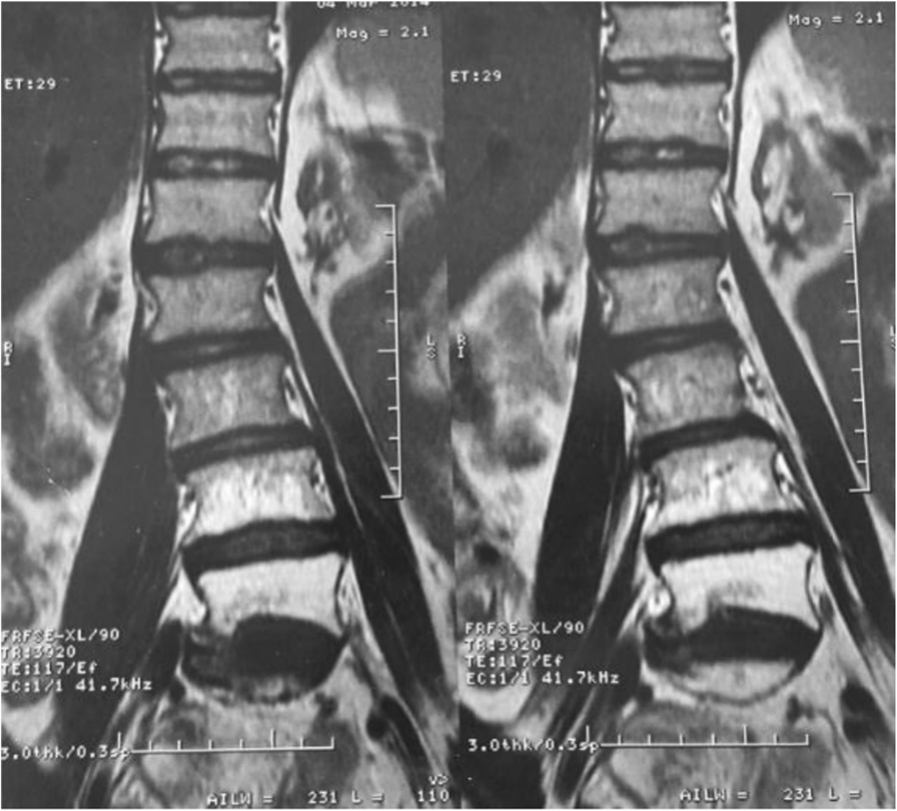
Spinal MRI T2-weighted sequence

Diagnostic studies including an abdominal-pelvic CT scan showed complete resolution of the primary tumor with no evidence of loco-regional recurrence. Cervical cytology was negative. Bilateral leg Doppler studies were normal. Magnetic resonance imaging (MRI) of the thoracic and lumbosacral spine did not show any pathologies, the T2 weighted MRI images revealed only increase in signal intensity in the radiated segment of cord suggestive of reactive lesion (Figs. 1, 2 and 3).

Mobility problems and pain in the lumbosacral spine were slowly but steadily growing, the patient was treated with oral morphine, gabapentin and Vitamins B1–B6.

Electromyogram and nerve conduction studies revealed a bilateral abnormality consistent with L4, L5, S1, radiculopathy greater on the left side. Cerebrospinal fluid (CSF) analyses were negative with no evidence of malignancy, infectious, or inflammatory process. Complete blood count and serum chemistry showed no abnormality that explained her syndrome. Finally the diagnosis of radiation-induced lumbosacral plexopathy was made by exclusion.

Five months post-treatment the patient’s symptoms have stabilized without significant improvement in her lower extremity weakness, at the time of this report, she has improved 50 % of her subjective baseline and can now walk with crutches. She remains on gabapentin.

## Discussion

For a number of years, radio-chemotherapy has been a treatment of choice in cervical cancer patients, starting from stage IB2 [2]. Radiation induced Lumbosacral plexophaty (RILP) is a rare but severe complication; its frequency ranges from 0.3 % to 1.3 % [2, 3]; it is characterized by a latent period between radiation exposure and the development of symptoms, according to data from the literature, it may take even 30 years from the end of radiotherapy to occurrence of neurological symptoms [2, 4–6].

Predisposing factors and the exact mechanism have been not be clearly elucidated, but factors that can lower the spinal cord tolerance to ionizing radiation include advanced age, obesity, hypertension, diabetes, dyslipidemia [6–8]. In addition to factors related to radiotherapy treatment such as a large total dose (>50 Gy to plexus, >60 Gy to cranial nerves) [1, 5, 6, 8–10], large dose per fraction [1, 5, 6, 9, 11], heterogeneous high-dose distribution [12], hot spot high dose (field junctions) [1, 13], intracavitary radium source [1, 13].

Recently, it has been suggested that the use of combined treatment might also lower the spinal cord tolerance to radiotherapy [1, 2, 6, 8]. In this present case, the proposed ballistic included only an anteroposterior/posteroanterior fields to 46 Gy, leaving the plexus inside all the time, with an additional 15 Gy of brachytherapy. Unfortunately the dose that the lumbosacral plexus received has not been determined.

On the other hand, some authors [1, 2, 6, 8] draws attention to the use of cisplatin in the treatment of cervical cancer with known secondary peripheral neurotoxicity, our patient received five courses of cisplatin; therefore, it remains to be determined whether the concomitant use of cisplatin with radiation will result in an increase in this neurological complication in our case.

RILP pathogenesis has been highly debated; nerve compression by indirect extensive radiation-induced fibrosis plays a central role; Schultheiss et al. [14, 15] suggest that injury to blood vessels especially venous by ischemia following capillary network failure remains the critical event.

Delanian et al. [1] defined this complication as a dynamic process related to perturbations at various levels of physiological homeostasis, characterised by gradual stepwise worsening over a period of several years, with the early changes consisting of spongy demyelination and axonal swelling, followed by atrophy, vascular damage, and plasma cell infiltrate in the latter stages.

Usually the symptoms begins with moderate pelvic pain (50 % of patients) [5, 9], the onset of neurological signs is insidious, with damage that is largely motor in the form of strong, persistent pain and reduced muscular strength, this symptoms are usually bilateral and asymetric with initial unilateral damage [1, 5, 9, 16]; the sensory signs and paraesthesia are absent or noted very late [1, 5]. Our case correspond to this clinical presentation, as well the first neurological symptoms, manifesting as progressive bilateral leg pain and lower extremity weakness, greater on the left side, with sensory changes over the lateral legs and feet.

Lumber magnetic resonance imaging (MRI) represents the first line exam; it does not play a determinant role in a positive diagnosis, but does eliminate tumour invasion or lumbar canal stenosis [8, 15]. Cerebrospinal fluid (CSF) evaluations may detect an elevated CSF protein; however, CSF is often negative for malignant cells, infection, or evidence of multiple sclerosis [6, 8, 15].

The diagnosis of RILP is essentially one of exclusion, metastasis or a local tumor progression with secondary nerve involvement usually leads the differential diagnosis, the minimal workup must include cerebrospinal fluid analysis, electromyography, and CT or MRI scans of the spinal cord [6, 8].

In all, three criteria have been proposed to retain the diagnosis of RILP: a history of radiotherapy in which the spinal cord was included within the field of radiotherapy, the main neurologic lesion is within the segments of the cord exposed to radiotherapy, and metastatic and other primary spinal cord diseases have been ruled out [6, 15].

No effective treatment for RILP exists; it has been known that combined pentoxifyllin-tocopherol (PE) significantly reduces symptoms due to their synergistic clinical and biological properties [17, 18]. Recently, Clodronate (a bisphosphonate, inhibits osteoclastic bone destruction with anti-inflammatory effects, and inhibits macrophagic myelin nerve destruction in rats [19]) when combined with pentoxifylline-tocopherol (PENTOCLO), healed 54 patients with refractory osteoradionecrosis in a median of 9 months [20]. Moreover, neurological symptoms were reduced by half in two patients with progressive RILP after 3 years of PENTOCLO treatment, results that have led to an ongoing phase III randomised clinical trial in France (NCT01291433) [1].

Symptomatic treatment includes conventional analgesics for pain, benzodiazepines for paraesthesia, quinine for cramps and membrane-stabilising drugs (carbamazepine) for reducing nerve hyperexcitability. Vitamins B1–B6 are often used routinely, but detailed data are lacking. Physical therapy is valuable in maintaining function and preventing joint complications [1, 2, 6, 8, 15].

In today’s clinical practice, the best approach is always prevention in respect of radiotherapy limits by reducing total dose, dose per fraction and volume every time if possible, while identifying patients with serious comorbidities [1, 15].

## Conclusion

RILP is a rare but devastating complication that has a considerable impact on quality of life in patients considered to be cured of their cervical cancer, Improved understanding and earlier diagnosis of this complication, before the lesions become progressive and irreversible, is particularly important, and more surveillance will be needed to assess the true incidence of this rare complication, its evolution and the best treatment to relieve it.

### Consent and statement of ethical approval

Patient’s treatment was decided by the medical staff of the centre; written consent was obtained from the subject and was approved by the institutional review boards of the National Institute of Oncology, Cancer Centre in Rabat.

## References

[1] Delanian S, Lefaix J-L (2012). Radiation-induced neuropathy in cancer survivors. Radiother Oncol.

[2] Klimek M, Kosobucki R (2012). Radiotherapy-induced lumbosacral plexopathy in a patient with cervical cancer: A case report and literature review. Contemporary Oncol.

[3] Topkan E, Onal HC, Yavuz AA, Yavuz MN (2008). Pathophysiology and management of radiation induced lumbosacral plexopathy. Turk onkoloji dergisi.

[4] Stryker JA, Sommerville K (1990). Sacral plexus injury after radiotherapy for carcinoma of the cervix. Cancer.

[5] Jousse M, Verollet D, Le Breton F (2010). Neuroperineal complications of pelvic radiotherapy. Pelvi-Périnéologie.

[6] Jeffrey DBLOSS, PHILIP J (1991). DISAIA. Radiation Myelitis: A Complication of Concurrent Cisplatin and 5Fluorouracil Chemotherapy with Extended Field Radiotherapy for Carcinoma of the Uterine Cervix. Gynecol Oncol.

[7] Delanian S, Lefaix J-L (2004). The radiation-induced fibroatrophic process: therapeutic perspective via the antioxidant pathway. Radiother Oncol.

[8] Robert VHIGGINS, VERESA T (1997). Radiation Myelopathy after Chemotherapy and Radiation Therapy for Fallopian Tube Carcinoma. Gynecol Oncol.

[9] Abu-Rustum NR, Rajbhandari D (1999). Acute Lower Extremity Paralysis Following Radiation Therapy for Cervical Cancer. Gynecol Oncol.

[10] Maier JG, Perry R, Saylor W, Sulak M (1969). Radiation myelitis of the dorsolumbar spinal cord. Radiology.

[11] Johansson S, Svensson H, Denekamp J (2002). Dose response and latency for radiation induced fibrosis, edema, and neuropathy in breast cancer patients. Int J Radiat Oncol Biol Phys.

[12] Delanian S, Pradat P-F (2010). A posteriori conformal radiotherapy using 3D dosimetric reconstitution in a survivor of adult-onset Hodgkin’s disease for definitive diagnosis of a lower motor neuron disease. J Clin Oncol.

[13] Ashenhurst EM, Quartey G, Starreveld A (1977). Lumbo-sacral radiculopathy induced by radiation. Can J Neurol Sci.

[14] Schultheiss TE (2008). The radiation dose response of the human spinal cord. Int J Radiat Oncol Biol Phys.

[15] Rafai MA, Boulaajaj FZ, Amriss O (2009). Radiation myelopathy. J Radiol.

[16] Georgiou A, Grigsby PW, Perez CA (1993). Radiation induced lumbosacral plexopathy in gynecologic tumors: clinical findings and dosimetric analysis. Int J Radiat Oncol Biol Phys.

[17] Delanian S, Porcher R, Balla-Mekias S, Lefaix J-L (2003). Randomized placebo controlled trial of combined pentoxifylline and tocopherol for regression of superficial radiation-induced fibrosis. J Clin Oncol.

[18] Hamama S, Gilbert-Sirieix M, Vozenin M-C, Delanian S (2012). Radiation-induced enteropathy: molecular basis of pentoxifylline-vitamin E antifibrotic effect involved TGF-b1 cascade inhibition. Radiother Oncol.

[19] Delanian S, Lefaix J-L (2007). Current management for late normal tissue injury: radiation induced fibrosis and necrosis. Semin Radiat Oncol.

[20] Delanian S, Chatel C, Porcher R, Depondt J, Lefaix JL (2011). Complete restoration of refractory mandibular osteoradionecrosis by prolonged treatment with a pentoxifylline– tocopherol–clodronate combination (PENTOCLO): a phase II trial. Int J Radiat Oncol Biol Phys.

